# Transient Bilateral Fixed Dilated Pupils Following Papaverine Use During Intracranial Microvascular Decompression: A Case Report

**DOI:** 10.7759/cureus.112127

**Published:** 2026-07-06

**Authors:** Shajahan Idayathulla, Shoaib Nawaz, Nissar Shaikh, Mansoor Nainthramveetil, Rahman MA, Fernando R Gonzalez

**Affiliations:** 1 Surgical Intensive Care Unit, Hamad General Hospital, Doha, QAT; 2 Anesthesiology, New York Medical College, Metropolitan Hospital Center, New York, USA

**Keywords:** cerebral vasospasm, fixed dilated pupils, microvascular decompression (mvd), open craniotomy, papaveriine

## Abstract

Fixed dilated pupils at the end of neurosurgical procedures are typically considered an ominous sign, often indicating acute brain injury, brainstem compression, or herniation, and are associated with poor neurological outcomes. However, pharmacological causes must also be considered. Papaverine hydrochloride, a vasodilator commonly used during intracranial surgery for the management of cerebral vasospasm, has been rarely reported to cause transient pupillary dilation and fixation. We describe the case of a 48-year-old woman with hemifacial spasm who underwent retrosigmoid craniotomy and microvascular decompression of the left facial nerve. The intraoperative course was uneventful under total intravenous anesthesia. Papaverine (200 mg) was locally infiltrated to relieve vasospasm of the anterior inferior cerebellar artery. At the conclusion of surgery, bilateral fixed dilated pupils (7 mm, nonreactive) were observed, raising concern for catastrophic intracranial pathology. Urgent neuroimaging was planned. However, within 15 minutes, the patient developed spontaneous respiratory effort, responded to commands, and her pupils returned to normal size and reactivity. She was extubated uneventfully and subsequently made a complete recovery, being discharged home on postoperative day 6. This case highlights a rare but important confounding effect of papaverine infiltration during neurosurgery. Papaverine may induce transient mydriasis mimicking signs of devastating neurological injury. Awareness of this phenomenon is critical for anesthesiologists, neurosurgeons, and critical care teams to avoid unnecessary interventions and guide appropriate postoperative decision-making.

## Introduction

Neurological assessment at the end of neurosurgical procedures is essential for detecting acute complications and establishing postoperative baseline status. This assessment includes evaluation of consciousness, pupillary size, and pupillary reflexes. Bilateral dilated and unreactive pupils in this context typically suggest raised intracranial pressure portending impending herniation secondary to severe brain injury, stroke, or brainstem compression and are often associated with poor prognosis [1,2]. In the absence of mydriatic agents such as atropine, this sign usually prompts urgent neuroimaging.

Although rare, transient pupillary dilation has been described following the use of papaverine hydrochloride during neurosurgery. Papaverine is a smooth muscle relaxant and vasodilator commonly administered intra-arterially, intracisternally, or by local infiltration to relieve vasospasm during aneurysm surgery and microvascular decompression [3,4]. A few reports describe papaverine-associated fixed and dilated pupils that can mimic catastrophic intracranial pathology [5-8]. We report a rare case of transient bilateral fixed dilated pupils following papaverine infiltration during microvascular decompression of the facial nerve.

## Case presentation

**Figure 1:**
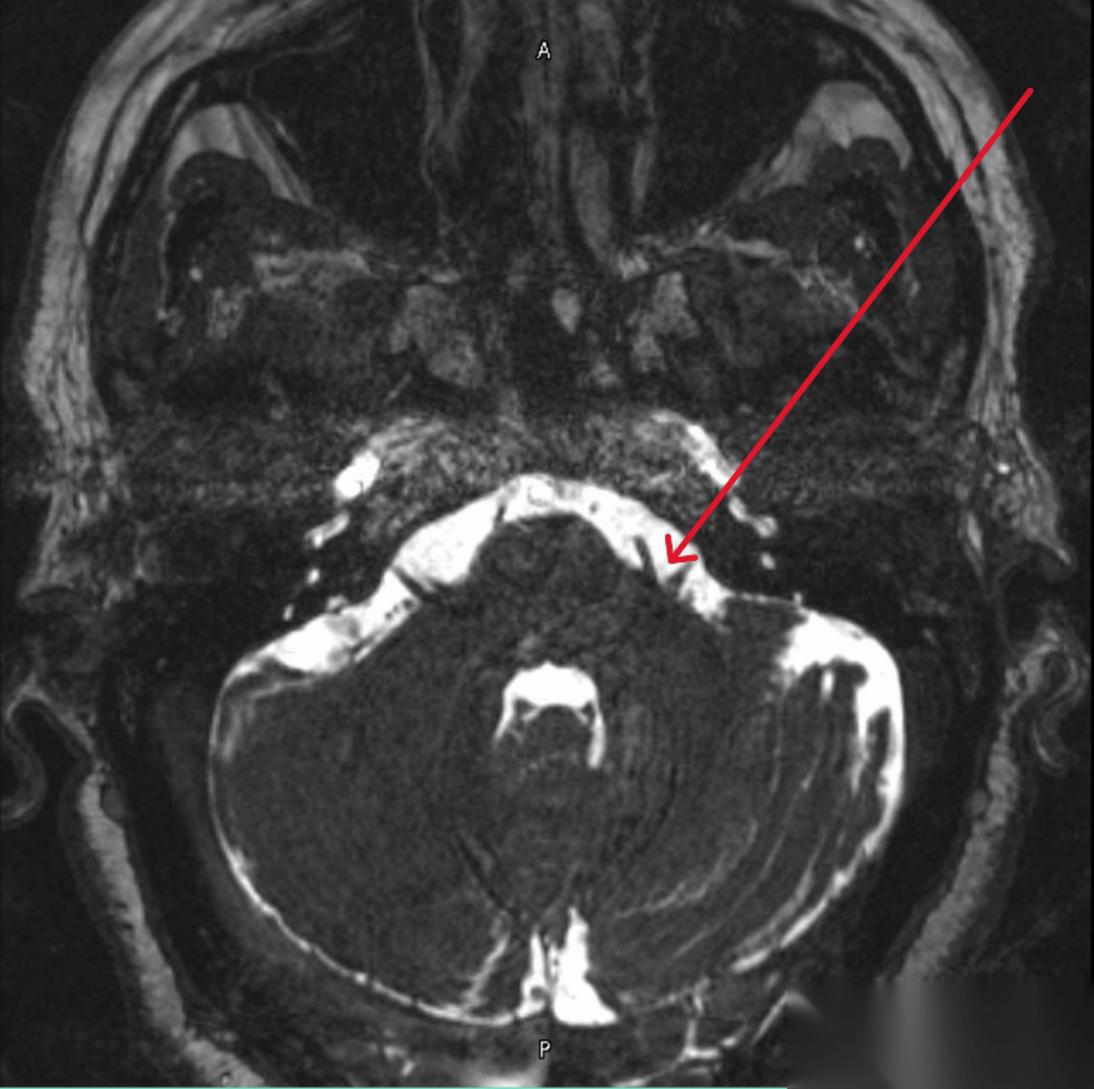
Axial high-resolution T2-weighted CISS MRI demonstrating an anomalous vascular loop of the left AICA in the left cerebellopontine angle, causing focal indentation of the cisternal segment of the left facial nerve at its root exit zone from the lower pons (arrow) AICA, anterior inferior cerebellar artery; CISS, constructive interference in steady state

A 48-year-old woman with no significant comorbidities presented with a long-standing history of left-sided hemifacial spasm involving the eye and ipsilateral facial muscles, associated with tinnitus and intermittent slurred speech. The patient had received several courses of botulinum toxin injections without relief. MRI revealed a vascular loop of the left anterior inferior cerebellar artery (AICA) indenting the cisternal segment of the left facial nerve (Figure 1). No cerebellopontine angle (CPA) mass or enhancing lesion was identified. Magnetic resonance angiography was otherwise unremarkable. A left retrosigmoid craniotomy and microvascular decompression of the left facial nerve was planned.

Intraoperative course

General anesthesia with total intravenous anesthesia was used. Scalp block was performed with 20 mL of 0.25% levobupivacaine. Induction was achieved with propofol (2 mg/kg), remifentanil (target-controlled infusion, 3-5 ng/mL), and rocuronium (1 mg/kg). The trachea was intubated orally using direct laryngoscopy. Standard monitoring was employed, including arterial line, bispectral index, and neuromuscular transmission monitoring. Maintenance was achieved with target-controlled infusions of propofol and remifentanil.

The patient remained hemodynamically stable throughout the 5-hour surgery. Blood pressure was supported with a minimal phenylephrine infusion (0.5-1 mg/hour). Analgesia included 1 g paracetamol and 3 mg morphine. Mannitol (50 g) and dexamethasone (8 mg) were administered for brain relaxation. Estimated blood loss was 200 mL; 1,500 mL of crystalloids were infused.

Surgical findings included compression of the facial nerve by a loop of the AICA. The vessel was dissected free, and Teflon felt was placed between the artery and nerve. During dissection, vasospasm of the AICA was observed and 200 mg of 0.3% papaverine was injected as direct focal application within the CPA. After hemostasis, the dura and wound were closed in layers.

Postoperative events

At the conclusion of surgery, anesthetic infusions were discontinued. Neurological assessment revealed bilaterally fixed dilated pupils (7 mm, nonreactive). The patient was unresponsive to verbal and painful stimuli, with Glasgow Coma Scale (GCS) E1M1VT, and minimal spontaneous respiration. This raised concern for acute brain injury. The surgical team was alerted, head of the bed was elevated to around 30 degrees to promote venous drainage from the head, 50 g (250 mL) of 20% IV mannitol bolus over 20 minutes was started, and blood pressure control with labetalol 20 mg IV bolus followed by titrated infusion was started to optimize cerebral perfusion pressure, as the patient was hypertensive following stoppage of anesthetic agents. An urgent CT scan was arranged.

Within 15 minutes, the patient coughed on the endotracheal tube, regained spontaneous ventilation, and responded to commands. Pupils returned to 3 mm and were reactive bilaterally. A repeat neurological examination revealed a GCS score of 15 with no motor or sensory deficit. Given the resolution of symptoms, CT imaging was deferred at that time.

**Figure 2:**
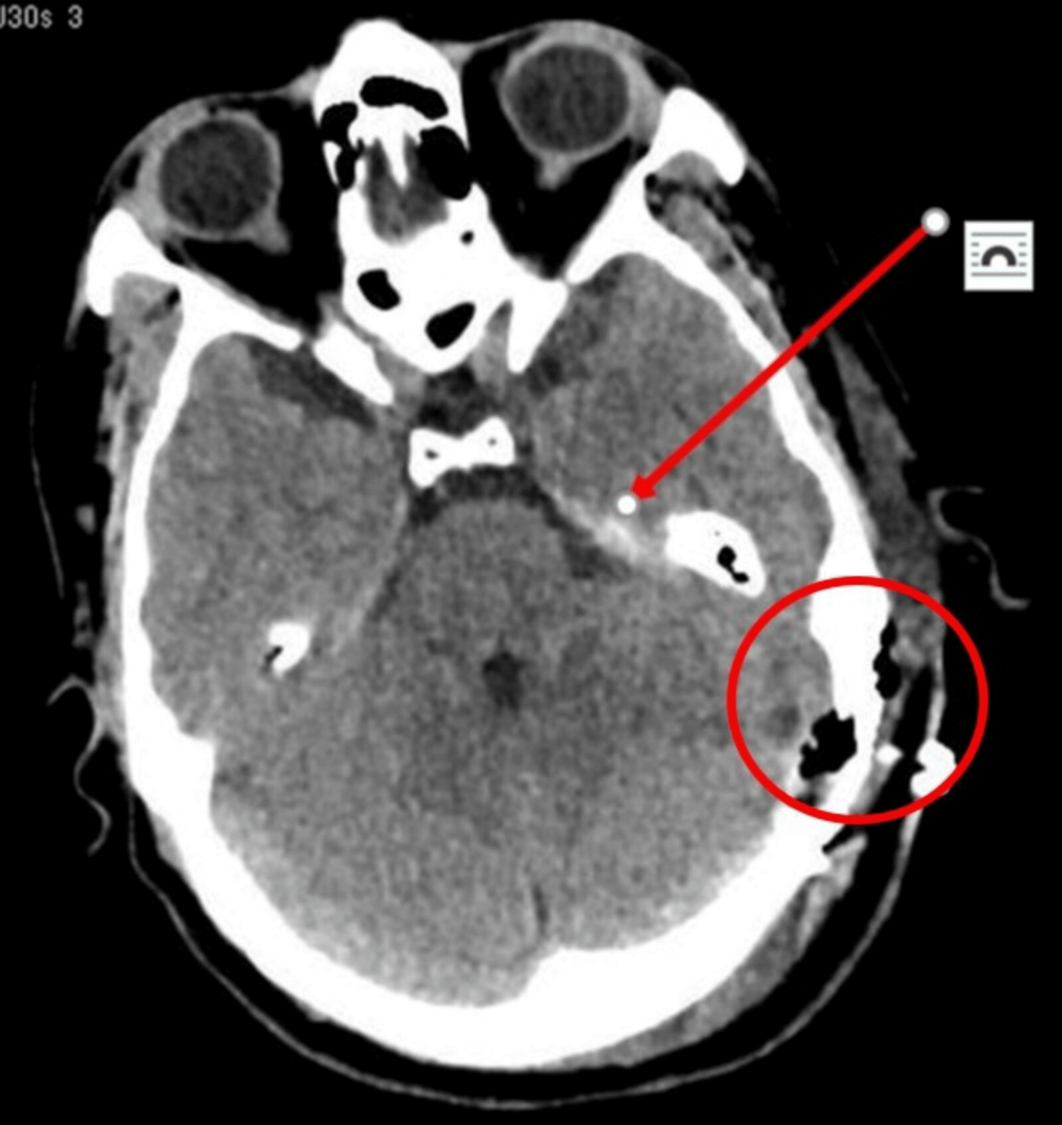
Axial CT scan of the head demonstrating the surgical incision site (red circle) and postoperative changes at the operative site with a focal hyperdensity (red arrow)

The patient was extubated uneventfully and transferred to the intensive care unit for observation. Postoperative recovery was uneventful. A follow-up CT scan performed the next day showed only postoperative changes (Figure 2). The patient was discharged home on postoperative day 6 with complete resolution of hemifacial spasm.

## Discussion

Fixed and dilated pupils in the perioperative setting are a red flag for the anesthesia and surgical teams, as they classically herald life-threatening intracranial events such as acute brainstem ischemia, herniation syndromes, or severe intracranial hypertension. In the absence of exogenous mydriatic drugs (e.g., atropine, sympathomimetics), pupillary fixation is considered an emergency finding that typically warrants urgent imaging and possible surgical re-exploration. Nevertheless, rare pharmacological effects, such as those induced by papaverine, must be considered in the differential diagnosis when the clinical context supports this explanation.

Pharmacology and mechanism of action

Papaverine is an opium alkaloid derivative that acts as a nonspecific phosphodiesterase inhibitor, thereby increasing intracellular cyclic AMP (adenosine monophosphate) and cyclic GMP (guanosine monophosphate) concentrations, leading to smooth muscle relaxation and vasodilation. Although its systemic administration can cause hypotension and cardiac depression, in neurosurgery, it is most often applied topically or instilled intracisternally for localized vasospasm relief [4,9].

The phenomenon of pupillary dilation following papaverine use is believed to result from its direct local effect on cranial nerve III. The oculomotor nerve carries parasympathetic fibers responsible for pupillary constriction, which run superficially on the dorsomedial aspect of the nerve. These fibers are particularly vulnerable to pharmacological blockades or ischemia. Diffusion of papaverine from the cisternal or perineural space through the fenestrated lamina terminalis and reaching the hypothalamus and brain stem can have a profound effect on the vital centers in the ventricular walls and can induce temporary paralysis of these fibers, leading to unopposed sympathetic input and subsequent mydriasis [4,10].

Additionally, papaverine has been shown to cause local irritation and even chemical meningitis when instilled in higher concentrations. The disruption of perineural conduction may contribute to transient cranial neuropathies, including reversible oculomotor dysfunction. Animal studies have also suggested that papaverine can impair mitochondrial respiration and calcium homeostasis in neural tissue, though these findings remain experimental [11,12].

Differential diagnosis

Intraoperative or immediate postoperative fixed dilated pupils may result from several critical etiologies including intracranial hemorrhage or infarction, especially if compressing the brainstem; acute herniation syndromes, particularly trans tentorial herniation; brainstem ischemia from vasospasm or vascular occlusion; hypoxic-ischemic brain injury due to intraoperative events; residual effects of neuromuscular blockers or anesthetics causing altered assessment (though these usually do not affect pupils directly); and drug-induced effects, such as topical mydriatics, scopolamine, atropine, or papaverine.

Recognition of papaverine as a reversible cause is critical, but given the high morbidity of the other causes, a cautious approach with urgent exclusion of catastrophic pathology remains mandatory [1].

Clinical implications for anesthesia and neurosurgery

For anesthesiologists, this phenomenon presents a diagnostic dilemma at a critical juncture - the transition from anesthesia to emergence. Premature assumptions may delay safe extubation, while unnecessary alarm may prompt emergent imaging or even re-exploration. Awareness of the temporal association between papaverine application and pupillary changes is key. Intraoperative communication between surgeons and anesthesiologists regarding papaverine administration should always be explicit.

In neurosurgery, especially during posterior fossa or CPA surgeries, papaverine is frequently applied in close proximity to cranial nerves. The risk of diffusion to the oculomotor nerve, although low, should be anticipated. Using lower concentrations, minimizing volume, and irrigating with saline after application have been suggested to reduce side effects [4].

Prognosis and reversibility

Unlike fixed dilated pupils due to structural brain injury, papaverine-induced pupillary changes are self-limiting and reversible, usually resolving within 15 minutes to 2 hours. Importantly, the reversibility of pupillary changes without neurological deficit is a distinguishing clinical feature. Documenting the onset, duration, and resolution of this effect can reassure the perioperative team and avoid unnecessary interventions [13].

Since the initial report by Pritz in 1994 [7], several reports have described similar cases. Al-Sharshahi et al. [4] documented significant hemodynamic effects after intracisternal papaverine instillation, while Sheshadri et al. [6] described three cases where transient fixed pupils after papaverine led to considerable diagnostic uncertainty. The rarity of these reports underscores the unusual but reproducible nature of this complication.

## Conclusions

Transient fixed dilated pupils following topical or intracisternal administration of papaverine represents a rare but clinically significant finding, which can potentially simulate catastrophic intracranial complications such as brainstem injury, intracranial hemorrhage, cerebral infarction, or transtentorial herniation. This case study underscores the need for awareness among clinicians to interpret this finding in the proper surgical context, during posterior fossa and CPA procedures, which may involve microsurgical application of papaverine in close proximity to cranial nerves. While immediate exclusion of structural causes remains imperative in this situation, rapid recovery of consciousness, spontaneous resolution of the pupillary dilatation, and the absence of other focal neurological deficits should help distinguish a true neurological catastrophe from this papaverine-induced phenomenon.
